# Structural Design and Sealing Performance Analysis of Biomimetic Sealing Ring

**DOI:** 10.1155/2015/358417

**Published:** 2015-06-02

**Authors:** Chuanjun Han, Han Zhang, Jie Zhang

**Affiliations:** School of Mechatronic Engineering, Southwest Petroleum University, Chengdu 610500, China

## Abstract

In order to reduce the failure probability of rubber sealing rings in reciprocating dynamic seal, a new structure of sealing ring based on bionics was designed. The biomimetic ring has three concave ridges and convex bulges on each side which are very similar to earthworms. Bulges were circularly designed and sealing performances of the biomimetic ring in both static seal and dynamic seal were simulated by FEM. In addition, effects of precompression, medium pressure, speed, friction coefficient, and material parameters on sealing performances were discussed. The results show that von Mises stress of the biomimetic sealing ring distributed symmetrically in no-pressure static sealing. The maximum von Mises stress appears on the second bulge of the inner side. High contact stress concentrates on left bulges. Von Mises stress distribution becomes uneven under medium pressure. Both von Mises stress and contact stress increase when precompression, medium pressure, and rubber hardness increase in static sealing. Biomimetic ring can avoid rolling and distortion in reciprocating dynamic seal, and its working life is much longer than O-ring and rectangular ring. The maximum von Mises stress and contact stress increase with the precompression, medium pressure, rubber hardness, and friction coefficient in reciprocating dynamic seal.

## 1. Introduction

The quality of sealing performance is one of the key indicators to estimate the properties of machineries. Seal failures, mainly caused by failures of sealing ring, would not only lower work efficiency but also lead to premature damage of machineries. Even worse, it may cause fire disaster, explosion, and so forth. For example, the Challenger space shuttle exploded because of a gap (10 mm) that appeared between the sealing ring and rigid body in 1986, which resulted in the leak of high temperature gas [1]. In the same year, Chernobyl disaster in Ukraine led to about 400 million people being affected by nuclear radiation for the leakage from reactors [2].

Rubber sealing rings are widely applied in machinery, petroleum industry, aerospace, and other fields for good sealing performance and low cost. The prevalent sealing rings include O-ring, rectangular ring, and X-ring. Generally, they can be used in static seal, reciprocating seal, and rotating seal. However, these sealing rings also have their disadvantages in dynamic seal. For instance, O-ring is prone to scroll and distort in reciprocating dynamic seal and these defects could lead to leak of medium. Although X-ring can replace O-ring reliably in many dynamic seal conditions, it is prone to fatigue failure. Rectangular ring is generally used only in static seal mainly because of the high friction caused by the large interaction face between sealing ring and the machine. Beyond that, it is difficult for rectangular ring to release the heat which would gather in the dynamic sealing process and the heat would damage the seal ability. So, it is essential to develop a kind of optimized sealing ring with the capacity of antidrag and better dynamic sealing performance.

Bionics was built on the research of the features of animal improved anatomies, functional skins, and plant structures. In order to face the survival challenges and adapt to the harsh environment, many creatures improved their anatomies and epidermis micromorphologies which made them survive. In recent years, with the development of manufacturing technology, it becomes possible to imitate the structures or functional skin of creatures.

More and more people research and explore in the field of biomimetic functional surface. For example, Gu carried out a theoretical analysis of bionic jet surface based on the shark gill slits and found out that bionic jet surface has remarkable drag reduction effect [3]. Inspired by skin structure of desert lizard, Huang et al. proposed a bionic sample which could improve the particle erosion resistance of engineering surfaces [4]. In addition, attempting to reveal the biologic features responding to skin friction drag reduction, the surface microstructure of fish scales was analyzed by Dou et al., and they found out that the proposed drag reduction technique shows the promise for practical applications [5].

So far, biomimetic sealing rings were mentioned in very few literatures. In this paper, biomimetic method was applied to design a new structure of sealing ring, and the sealing performances of the biomimetic ring in both static seal and dynamic seal were simulated by finite element method. This biomimetic sealing ring has better capacity of drag reduction and dynamic sealing performance than other sealing rings. In addition, the effects of precompression, working pressure, speed, friction coefficient, and material parameters on sealing performance were discussed.

## 2. Design of Biomimetic Sealing Ring

### 2.1. Properties of Nonsmooth Surface

**Figure 1:**
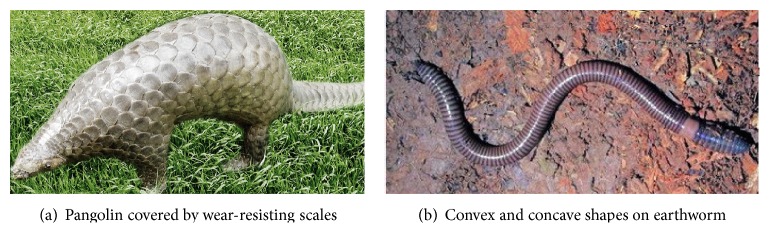
Nonsmooth surface of creatures.

After 3.5 billion years of evolution, many creatures have developed their smooth surface into nonsmooth one with the capacity of antidrag to seize the survival chance [6]. For example, lotus leaf has the self-cleaning function for its nonsmooth surface covered with micron-sized mastoid portions. Covered by placid scales which reduce the resistance of water, shark's skin makes it possible to swim at the amazing speed of 60 Km/h [7]. The surface of pangolin is covered by scales which are wear-resisting (Figure 1(a)). Owing to the convex shape bulges and the concave shape ridges on its surface, earthworm could get in and out of the soil without adhering to the mud (Figure 1(b)).

According to the growth mechanism of organisms and all the laws of nature [8], scientists have established morphological and structural bionics, also based on engineering practice. Using nonsmooth surface to reduce resistance is very ecofriendly, because it does not need additional devices or cause more waste. According to this theory, animals that live in different environment have different construction units on nonsmooth surface, including squamous shape, convex, concave, and ripple [9].

Living in soil, earthworm has extraordinary body structure which is composed of much similar somite. From outward appearance, earthworm looks very slender and like cylinder in shape. Concave can not only reduce contact area between itself and soil but also store excretive lubricating fluid (secreted from dorsal pores) to keep itself moist and reduce drag.

### 2.2. Structure Design

**Figure 2:**
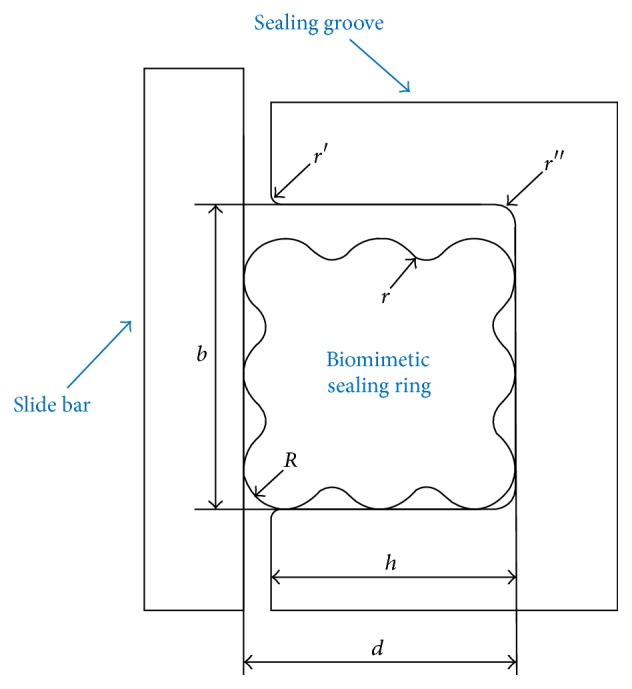
Schematic diagram of biomimetic sealing ring.

Inspired by functional surface of earthworm, morphological and structural bionics were used in the design of sealing ring. As shown in Figure 2, a new biomimetic sealing ring was designed based on O-ring, rectangular ring, and X-ring. There are three concave ridges and three convex bulges on each side of the biomimetic ring, which is very similar to the surface of earthworm. In order to take advantages of the O-ring-like homogeneous stress distribution, all the bulges were designed as circular. Resembling X-ring, four corners of biomimetic sealing ring are circular arc transition.

This new structure has many advantages as follows.

1. Under lubrication condition, concave can serve as fluid dynamic bearing to generate additional fluid dynamic pressure.
2. When concave has enough volume, it can store lubricants and lubricate tribopair in dynamic seal.
3. Concave has the capacity of storing abrasive impurities to lower the abrasion caused by particles.
4. Under action of precompression and medium pressure, the bulges can achieve self-sealing very well and three sealing tapes work in the main sealing surface, which can ensure excellent sealing performance.
5. In reciprocating dynamic seal, biomimetic sealing ring could avoid rolling and distortion and the working life of the ring could be prolonged.
6. Due to the smaller contact area between biomimetic sealing ring and rigid wall, not only friction and energy consumption could be reduced but also work efficiency could be improved.

## 3. Finite Element Model

### 3.1. Material Constitutive of Rubber

Modelling of this rubber design implies several nonlinearities such as geometrical, contact interaction, and material behavior. Rubber can be modeled as a kind of hyperelastic material by means of varied panoply of constitutive models such as Heo-Hookean strain energy function, Exponential-Hyperbolic rule, Mooney-Rivlin model, Klosenr-Segal model, and Ogden-Tschoegl model. In this paper, the Mooney-Rivlin model was selected to describe the mechanical characteristics of rubber linings. The function can be expressed as follows [10]:

(1) $$\begin{array}{c} W=C_{1}\left(\;I_{1}-3\;\right)+C_{2}\left(\;I_{2}-3\;\right), \end{array}$$

where *W* is the strain energy density, *C*_1_, *C*_2_ are Mooney-Rivlin coefficient, and *I*_1_, *I*_2_ are the first and second strain tensor invariant.

The relationship of stress and strain can be expressed as follows:

(2) $$\begin{array}{c} \sigma =\frac{\partial W}{\partial \varepsilon }. \end{array}$$

It is confirmed that material constants of Mooney-Rivlin model are related to the linear elastic modulus *G*, and *G* can be expressed as follows [11]:

(3) $$\begin{array}{c} G=2\left(\;C_{1}+C_{2}\;\right). \end{array}$$

According to the rubber compression test, *C*_1_ = 1.87 and *C*_2_ = 0.47. The density of rubber *ρ* = 1200 kg/m^3^.

### 3.2. Experiment of Friction Coefficient

**Figure 3:**
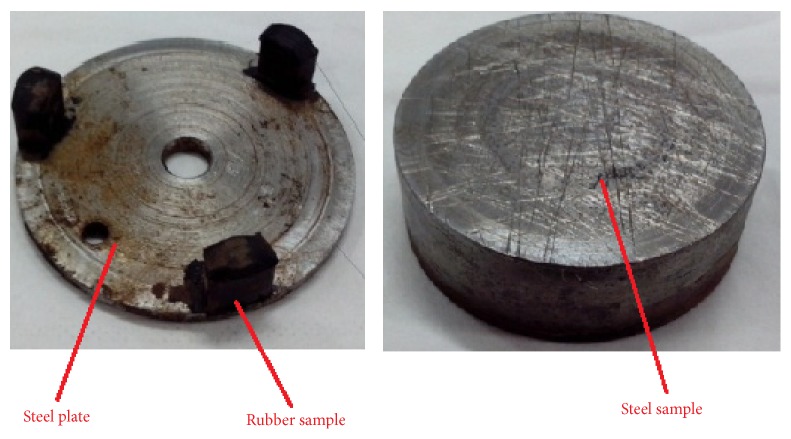
Samples of rubber and steel.

The friction coefficient between rubber and steel was tested using MMW-1 friction testing machine (Jinan Caide Instrument Co., Ltd.). The rubber samples were fixed on the steel plate by vulcanization as shown in Figure 3. The hardness of rubber is 80 Hr, tensile strength is not less than 16 MPa, tensile elongation is not less than 200%, and volume change rate is less than 15%. The material of steel sample is medium carbon quenched and tempered steel. Steel sample is cylindrical. The friction coefficients between rubber and steel sample under different lubricating conditions, including no lubricant condition, water lubrication condition, oil lubrication condition, water-base mud condition, oil-base mud condition, and oil-base lubricant condition, were tested when axial compressive force (*F* = 30 N) was loaded on.

**Figure 4:**
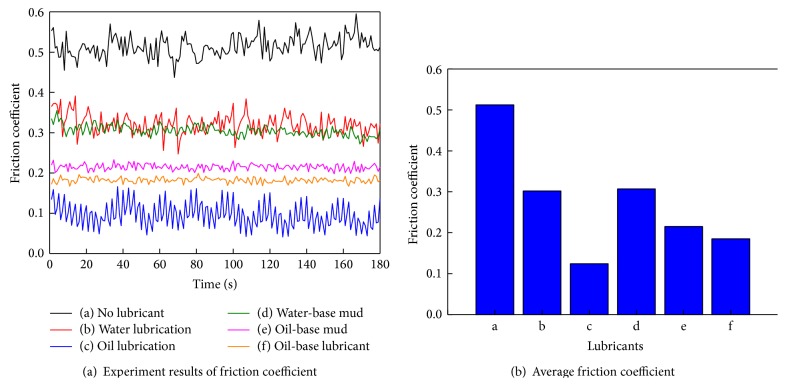
Experiment results of friction coefficients under different lubrication media.

Figure 4 shows curves of the friction coefficients between rubber and steel samples under different lubricating conditions. The friction coefficient changes with the change of lubricating condition. The maximum friction coefficient appears in no lubricant condition while the minimum friction coefficient appears in oil-base lubricant condition. Friction coefficients in water lubrication condition and water-base mud condition are approximately the same. The friction coefficient in oil-base lubricant condition is smaller than that in oil-base mud condition. In spite of different lubricating conditions, friction coefficient fluctuates up and down around a fixed value, respectively.

### 3.3. Geometric Model

The sealing performance of sealing ring was examined numerically by using advanced computational tools. Considering the nonlinear geometry of rubber material, a general purpose advanced finite element program (ABAQUS 6.11) was applied to simulate the stress and strain of rubber ring in a rigorous manner. Two-dimensional axisymmetric finite element models of biomimetic ring, groove, and slide bar were established based on the actual structure of the sealing system. According to the specifications, section width of the biomimetic sealing ring is 5.33 mm. The materials of groove and slide bar are both medium carbon quenched and tempered steel whose density is 7800 kg/m^3^, Poisson's ratio is 0.3, and modulus of elasticity is 210 GPa. As shown in Figure 2, cross-sectional diameter of the biomimetic ring *d* = 5.33 mm, radius of convex bulge *R* = 0.8 mm, and radius of the concave ridge *r* = 0.43 mm. Besides, depth of the groove *h* = 4.80 mm, width of the groove *b* = 6 mm, chamfer *r*′ = 0.2 mm, and *r*′′ = 0.4 mm.

**Figure 5:**
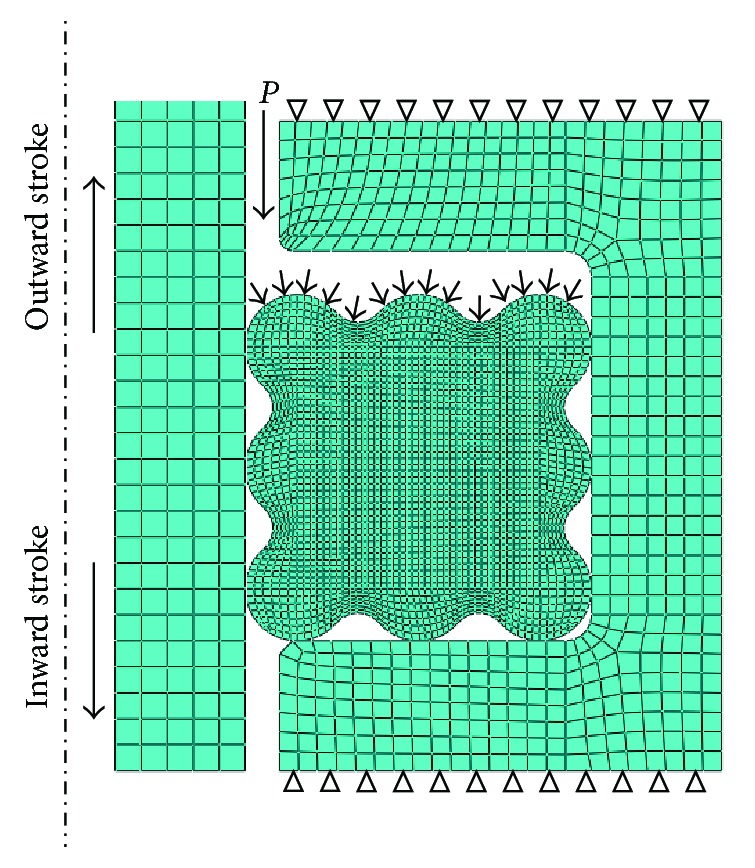
Finite element models.

A contact penalty algorithm with a friction coefficient equal to 0.3 was employed to simulate the interactions between the ring and steel material. In the current study, a contact algorithm based on contact pairs was defined between ring's surface and surface of groove and also between ring's surface and the surface of slide bar. As shown in Figure 5, four-node quadrilateral bilinear axisymmetric elements (CAX4R) were used for modeling all the bodies. The element size of biomimetic ring is 5 × 10^−5 ^m. Mesh sensitivity study was done by refining element size as 6 × 10^−5 ^m and 4 × 10^−5 ^m. Compared with 5 × 10^−5 ^m, the important parameters with 6 × 10^−5 ^m are a little smaller. The important parameters with 4 × 10^−5 ^m are very close to those with 5 × 10^−5 ^m. But computing time of FE model with 4 × 10^−5 ^m mesh is twice that with 5 × 10^−5 ^m mesh. Therefore, the FE model with 5 × 10^−5 ^m is reliable and time saving.

### 3.4. Fundamental Assumption

Since rubber has the material nonlinearity, geometrical nonlinearity, and contact nonlinearity, it is necessary for mechanical and sealing performance research to make the following assumptions.

1. Fluid medium has no corrosive effect on sealing rings.
2. Rubber sealing ring is not affected by medium temperature.
3. Creep does not affect the volume of the sealing ring.

### 3.5. Loading and Boundary Conditions

Sealing performances of static and reciprocating dynamic seal were researched. In accordance with actual conditions, static sealing process was achieved by two steps. Firstly, precompression (0.3 mm) was completed to simulate the installation process of sealing ring. Secondly, medium pressure (*P* = 3 MPa) was loaded on the working surface of sealing ring. Reciprocating dynamic sealing process was achieved by three steps. The former two steps are the same as above. The third step was to apply the axial velocity (*v* = 0.2 m/s) at slide bar. Outward stroke was defined as the slide bar moving against the pressure. On the opposite, when slide bar moves towards the same direction of the medium pressure, it was called inward stroke.

## 4. Static Sealing Performances

### 4.1. Stress of Sealing Ring

**Figure 6:**
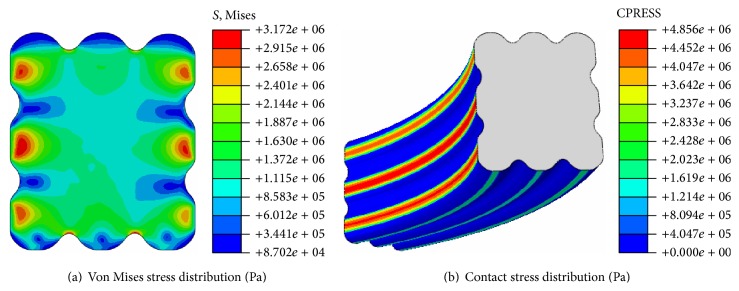
Stress distribution of sealing ring under no-pressure condition.

Stress distributions of the biomimetic sealing ring under no-pressure condition are shown in Figure 6. Von Mises stress of the ring is distributed symmetrically with respect to a center line of the cross section (as seen in Figure 6(a)). The maximum von Mises stress is 3.72 MPa, and it appears on the second bulge of the inner side. Von Mises stress distribution of the biomimetic sealing ring agrees with Hertz contact theory that stress does not appear on the contact surface but around where inside the ring. As shown in Figure 6(b), the maximum contact stress is 4.856 MPa and high contact stress is concentrated on three left bulges which also were called the main sealing surface. Since medium pressure is 0 MPa, contact stress of the bottom of the sealing ring is very small.

**Figure 7:**
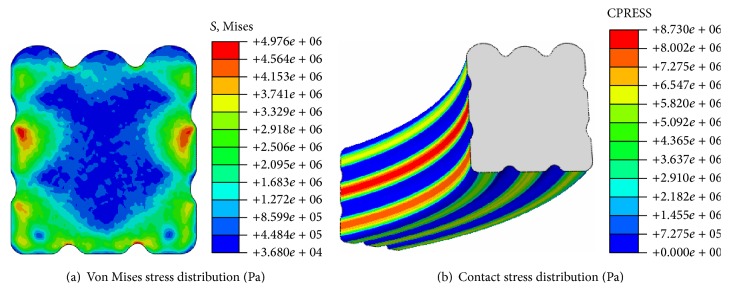
Stress distribution of sealing ring when *P* = 3 MPa.

When medium pressure is 3 MPa, stress distribution of the biomimetic sealing ring is as shown in Figure 7. The contact stress between the ring and rigid body increased after the ring was compressed by medium pressure. In other words, self-seal of the ring has been achieved by medium pressure. Von Mises stress of the bottom was increased and the stress distribution became more uneven with the increasing of medium pressure. The maximum von Mises stress is 4.976 MPa which is about 1.804 MPa higher than it in no-pressure condition. The maximum von Mises stress still appears on the second bulges although medium pressure plays an important role in this sealing condition. Meanwhile, the maximum contact stress (8.73 MPa) appears on the main sealing surface as under no-pressure condition. According to the criteria, the maximum contact stress has to be greater than or equal to the medium pressure to meet the requirements of sealing; otherwise it may cause leakage. Therefore, this paper mainly focused on the stresses of the main sealing surface.

**Figure 8:**
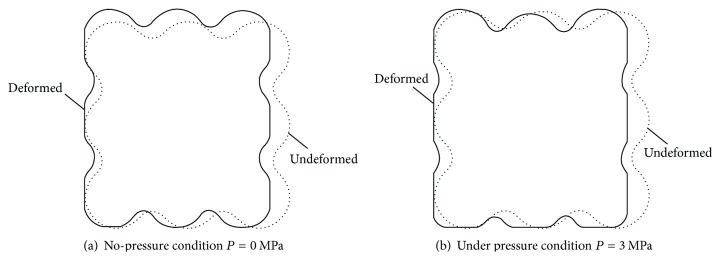
Deformation image of sealing ring.

Figure 8(a) shows the deformation of sealing ring under no-pressure condition. Under the action of precompression, the sealing ring is squeezed, and its height increases by 0.288 mm along the axial.  Figure 8(b) shows the deformation of sealing ring when medium pressure is 3 MPa. Since the action of medium pressure offsets the action of radial precompression, axial deformation of sealing ring is small, which is only 0.09307 mm.

### 4.2. Precompression Effect

**Figure 9:**
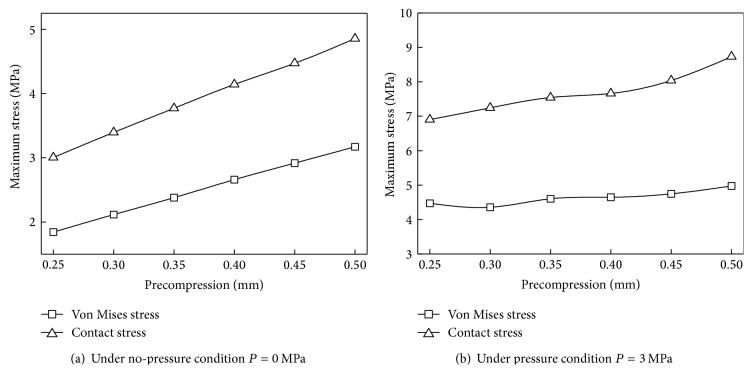
Stress of the biomimetic sealing ring under different precompression.

Appropriate precompression is an essential factor for sealing ring to achieve stable and reliable self-tightening seal.  Figure 9 shows the maximum von Mises stress and contact stress of the biomimetic ring under different precompression when *P* = 0 MPa and *P* = 3 MPa. In both no-pressure condition and pressure condition, the von Mises stress and contact stress increase with the increasing of precompression. Two kinds of stress under no-pressure condition are growing linearly, but nonlinearly under pressure condition. The compression-stress curves of the ring present fluctuations with small amplitude under pressure, but the growth rate of von Mises stress is smaller. It means that precompression has a smaller effect on the von Mises stress under pressure condition than under no-pressure condition, because axial strain caused by medium pressure can resist radial strain caused by precompression.

### 4.3. Friction Coefficient Effect

**Figure 10:**
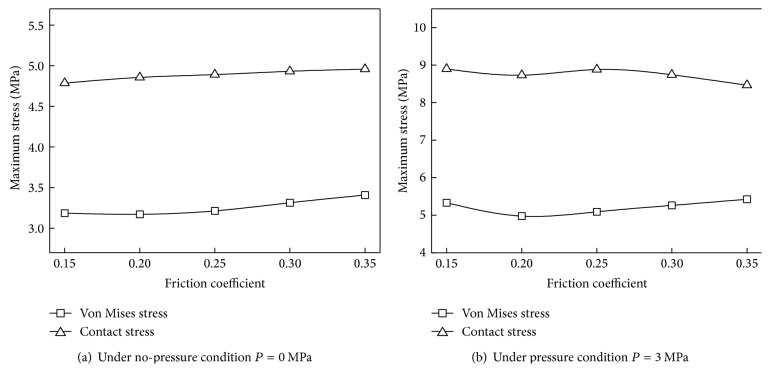
Stress of the biomimetic sealing ring under different friction coefficients.

According to the experimental result, friction coefficient under different lubricating conditions is different. The structure of the biomimetic sealing ring was designed to store the lubricating fluid so that all the working conditions should be postulated as lubricated. Numerical simulations with friction coefficient range from 0.15 to 0.35 were investigated, and the stress distributions are shown in Figure 10. Under no-pressure condition, both von Mises stress and contact stress increase with the increasing of friction coefficient, but within a small margin. As is shown in Figure 10(b), when medium pressure is loaded, biomimetic ring's contact stress reduces with the increasing of friction coefficient, which means that the sealing performance has been weakened but still could meet the sealing requirement. However, von Mises stress first decreases and then increases with the increasing of friction coefficient.

### 4.4. Medium Pressure Effect

**Figure 11:**
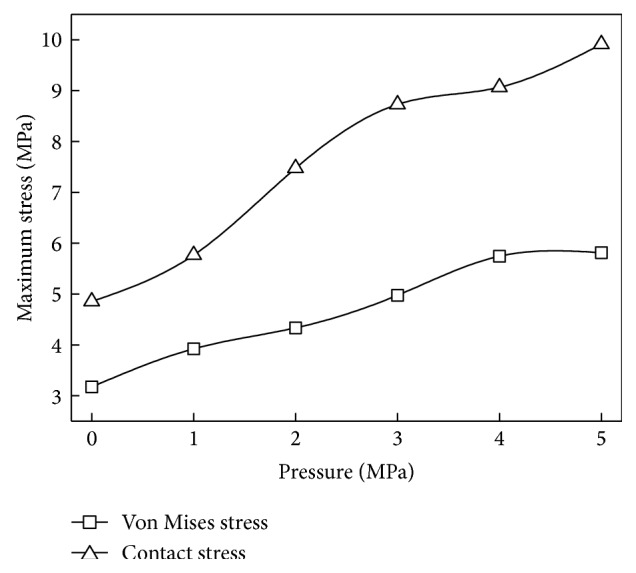
Stress of the biomimetic sealing ring under different medium pressures.

Figure 11 shows the curves of the maximum von Mises stress and contact stress under different medium pressures. Both von Mises stress and contact stress increase with the pressure but grow nonlinearly. The maximum contact stress of the main sealing surface is much higher than the medium pressure which makes it possible for biomimetic sealing ring to maintain good performance in static seal.

### 4.5. Rubber Material Effect

Except in rare and exceptional circumstances, the Shore hardness of rubber sealing ring is from 70 to 90 Hr. Through fitting many formulas, Liu derived physical parameters (*C*_1_ and *C*_2_ are Mooney-Rivlin coefficients and *E* is corresponding elasticity modulus) of rubber under different material hardness (shown in Table 1) [12]. The physical parameters are well consistent with the corresponding experimental ones.

**Figure 12:**
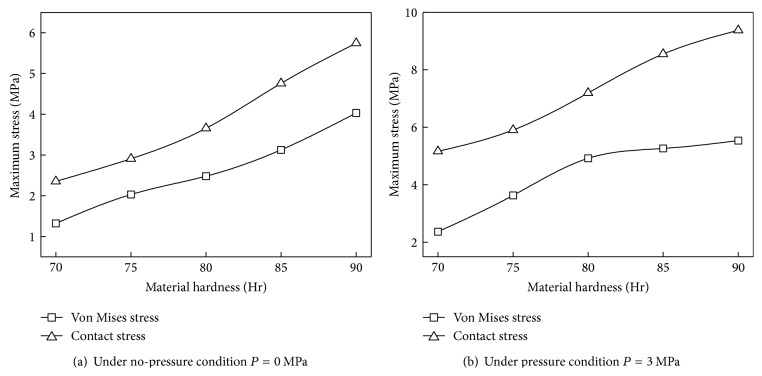
Stress of the biomimetic sealing ring under different material hardness.

Figure 12 shows the maximum stresses of the ring with different material hardness. In both no-pressure condition and pressure condition, the maximum contact stress increases nonlinearly and the sealing performance becomes better with the increasing of the hardness of rubber material.

Nevertheless, higher von Mises stress in two different conditions could result in premature failure of the sealing ring. When *P* = 3 MPa, the growth rate of von Mises stress and contact stress reduces gradually with the increasing of the hardness.

## 5. Reciprocating Dynamic Sealing Performances

### 5.1. Comparison with Other Sealing Rings

**Figure 13:**
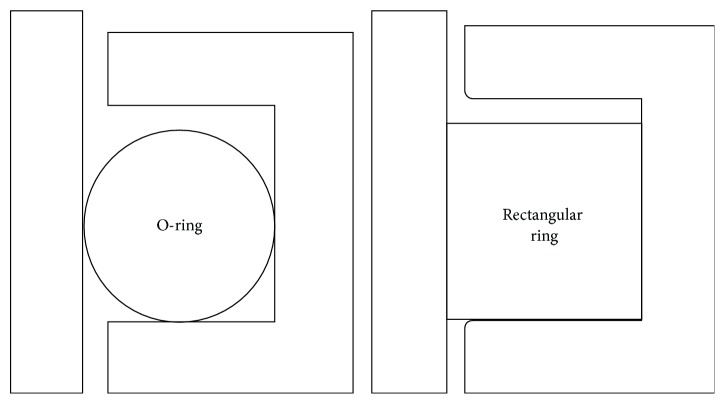
Schematic diagrams of O-ring and rectangular ring.

In order to research reciprocating dynamic sealing performance of the biomimetic sealing ring, the sealing performance is compared with other kinds of sealing rings. Schematic diagrams of O-ring and rectangular ring, which have the same size as biomimetic sealing ring, are shown in Figure 13. Reciprocating dynamic sealing processes of these three rings are simulated by finite element method as well.

**Figure 14:**
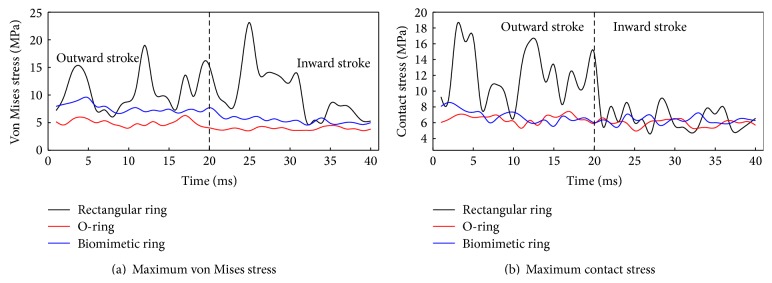
Stress of sealing rings in reciprocating dynamic seal.

The maximum von Mises stress and contact stress of sealing rings mentioned above are shown in Figure 14. The maximum stress is fluctuant in the process for the viscoelasticity of rubber material. As shown in Figure 14(a), von Mises stress of rectangular ring is higher than O-ring and biomimetic ring, and its stress fluctuation is the biggest. It means that rectangular ring is prone to be torn or result in fatigue failure. Von Mises stress distributions of O-ring and biomimetic ring are more even in outward stroke and inward stroke. Therefore, biomimetic ring could avoid premature failure.

As shown in Figure 14(b), contact stress fluctuation of rectangular ring is more violent in outward stroke; serious creeping phenomenon appears. So, rectangular ring is not suitable for dynamic sealing. The contact stress of biomimetic ring and that of O-ring are approximately the same, as well as their variation tendencies. Therefore, biomimetic ring has the same sealing performance as O-ring, but biomimetic ring can avoid rolling and distortion in reciprocating dynamic seal. So, the working life of biomimetic is much longer than O-ring.

### 5.2. Precompression Effect

**Figure 15:**
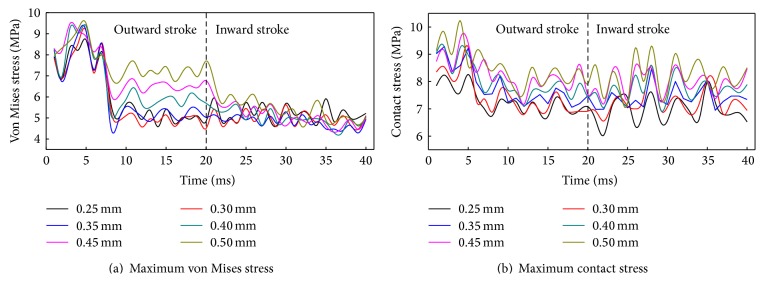
Stress of the biomimetic sealing ring under different precompression.

Figure 15 shows the maximum von Mises stress and contact stress of the biomimetic sealing ring under different precompression. Before 7 ms, the maximum static friction is overcome and reciprocating motion begins. The precompression has a small effect on stress fluctuation rule. As shown in Figure 15(a), the maximum von Mises stress increases with the precompression in outward stroke, but effect of the precompression on von Mises stress is small in inward stroke. As shown in Figure 15(b), the maximum contact stress increases with the increasing of the precompression. The contact stress is higher at the beginning of reciprocating motion. Contact stress fluctuation in inward stroke is higher than in outward stroke.

### 5.3. Friction Coefficient Effect

**Figure 16:**
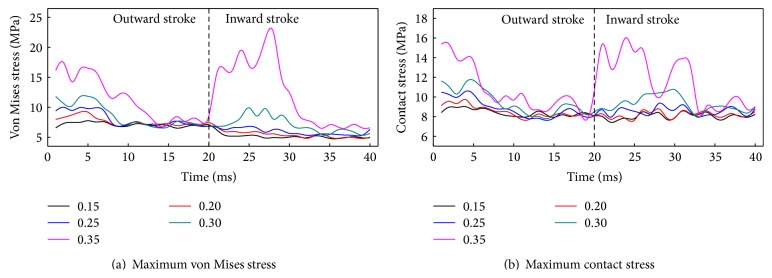
Stress of the biomimetic sealing ring under different friction coefficients.

Figure 16 shows the von Mises stress and contact stress of the biomimetic sealing ring under different friction coefficients. At the beginning of the reciprocating motion, the von Mises stress and contact stress are higher than those in the later reciprocating motion. Von Mises stress and contact stress increase with the increasing of friction coefficient, but the change rates in inward stroke are higher than that in the outward stroke. When friction coefficient is larger than 0.3, creeping phenomenon appears. Therefore, lubricating is important for reciprocating dynamic seal. The concaves of the biomimetic ring could store lubricant to ensure that the ring could be lubricated for a longer time.

### 5.4. Medium Pressure Effect

**Figure 17:**
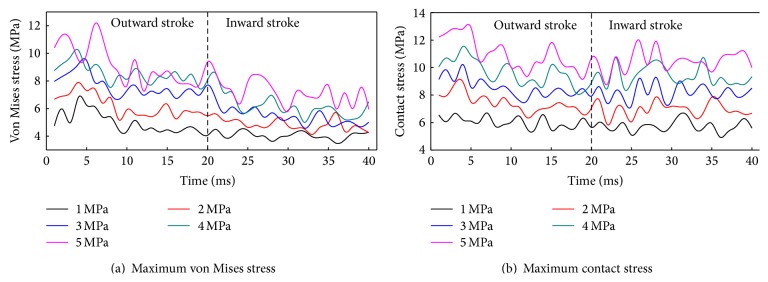
Stress of the biomimetic sealing ring under different medium pressures.

Figure 17 shows the von Mises stress and contact stress of the biomimetic sealing ring under different medium pressures. With the increasing of medium pressure, von Mises stress and contact stress increase gradually, but the stress fluctuation also increases. The maximum contact stress in main sealing surface of biomimetic ring is higher than medium pressure when *P* ≤ 5 MPa. Therefore, the reciprocating dynamic sealing performance of the biomimetic sealing ring is stable and reliable.

### 5.5. Rubber Hardness Effect

**Figure 18:**
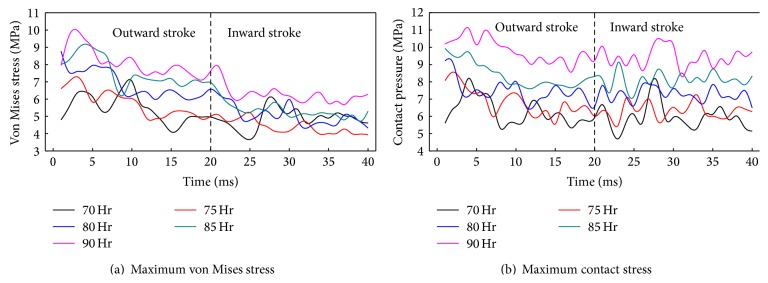
Stress of the biomimetic sealing ring under different material hardness.

Figure 18 shows von Mises stress and contact stress of the biomimetic sealing ring under different material hardness. As shown in Figure 18(a), with the increasing of material hardness, von Mises stress increases gradually. The von Mises stress in inward stroke is smaller than in the outward stroke. As shown in Figure 18(b), contact stress increases gradually with the increasing of material hardness. When material hardness is 70 Hr or 90 Hr, the stress of the biomimetic ring fluctuates most seriously. Therefore, it is essential for biomimetic ring to have a reasonable hardness to ensure good reciprocating dynamic sealing performance.

## 6. Conclusions

1. According to bionics, a new biomimetic sealing ring was designed based on O-ring, rectangular ring, and X-ring. There are three concave ridges and three convex bulges on each side of the biomimetic ring, and it is very similar to earthworms. All the bulges were designed as circular, and four corners of biomimetic sealing ring are circular arc transition.
2. In static sealing, von Mises stress of the biomimetic sealing ring distributes symmetrically under no-pressure condition. The maximum von Mises stress appears on the second bulge of the inner side. High contact stress is concentrated on the three left bulges which also are called the main sealing surface. Under medium pressure, distribution of von Mises stress becomes uneven.
3. In static sealing, both von Mises stress and contact stress increase with the increasing of precompression, medium pressure, and hardness of rubber material, but friction coefficient has a small effect on the stress of biomimetic sealing ring.
4. The maximum stress is fluctuant in the moving process for the viscoelasticity of rubber material. Von Mises stress fluctuation of rectangular ring is higher than both O-ring and biomimetic ring. Contact stresses of biomimetic ring and O-ring are approximately the same, and their variation tendencies are the same too, but biomimetic ring can avoid rolling and distortion in reciprocating dynamic seal. Therefore, working life of biomimetic ring is much longer than O-ring and rectangular ring.
5. In reciprocating dynamic seal, both the maximum von Mises stress and contact stress increase with the increasing of the precompression, medium pressure, rubber hardness, and friction coefficient. When friction coefficient is larger than 0.3, creeping phenomenon appears.

## Figures and Tables

**Table 1:** Physical parameters of different hardness.

Hardness [Hr]	*E* [MPa]	*C*_1_	*C*_2_
70	6.96	1.137	0.023
75	8.74	1.444	0.0165
80	10.98	1.833	−0.003
85	13.98	2.334	−0.034
90	17.33	2.972	−0.082
